# Biodiesel from Dodonaea Plant Oil: Synthesis and Characterization—A Promising Nonedible Oil Source for Bioenergy Industry

**DOI:** 10.3389/fbioe.2022.864415

**Published:** 2022-06-06

**Authors:** Syeda Alia Zehra Bukhari, Kifayat Ullah, Mushtaq Ahmad, Muhammad Zafar, Anwar Ullah, Shazia Sultana, Mohamed Mousa Ibrahim, Mahmoud Mohamed Hessien, Gaber Ahmad Mahmoud Mersal, Sherif Salama Mohamed Ghoneim

**Affiliations:** ^1^ Biofuel & Biodiversity Lab, Department of Plant Sciences, Quaid-i-Azam University, Islamabad, Pakistan; ^2^ Department of Biosciences, COMSATS University Islamabad, Islamabad, Pakistan; ^3^ Department of Chemistry, College of Science, Taif University, Taif, Saudi Arabia; ^4^ Department of Electrical Engineering, College of Engineering, Taif University, Taif, Saudi Arabia

**Keywords:** dodonaea plant, nonedible oil source, biodiesel, fuel properties analysis, spectroscopic studies

## Abstract

In this work, Dodonaea oil was studied as a potential biodiesel source. Dodonaea (*Dodonaea viscosa* Jacq.) is an evergreen shrubby plant that thrives in tropical and subtropical conditions. The plant produces high-grade biodiesel in terms of both quantity and quality despite its naturally high fat content. In the transesterification followed by esterification reaction, varied ratios of oil to methanol, constant temperature (60^°^), reaction duration (1 h), and different catalyst concentrations (0.25–0.75% (w/w) were utilized. A maximum biodiesel yield of 90% was achieved. For fuel characteristic analysis, the prepared biodiesel was specified and compared to ASTM criteria. The chemical composition was verified using analytical techniques such as FT-IR and NMR spectroscopy. As a result of the foregoing, Dodonaea is considered a possible bioenergy source, particularly in the transport sector.

## 1 Introduction

Traditional energy supplies such as natural gas, coal, and petroleum currently meet the increasing global energy demand, but these resources are rapidly decreasing and are at the point of extinction (Demirbas 2007; Mahmood et al., 2014). Due to the uncertainty of fossil-based fuels and mounting environmental harms, the world’s interest in alternative, clean, and sustainable energy options has shifted (Barnwal and Sharma 2005).

The primary sources of electricity are petroleum-based fuels such as oil, coal, and natural gas. However, as humanity’s reliance on fossil fuels has grown, the rate of asset exhaustion and the threat of global warming have also increased. As a result, the issue persists; alternative fuels must be developed to reduce fossil fuel consumption, while also reducing greenhouse gas emissions. In this circumstance, biomass derivative fuels, which are renewable, sustainable, and environmentally benign, may be a superior option.

Biodiesel plays a key role in reducing greenhouse gas emissions in the transportation sector, and the European Union is increasing its relevance for sharing, which is equivalent to an emission reduction of nearly 85% in this sector. Few actions are required to achieve sufficient progress in the transportation area, given the existing circumstances of strong political support and market expansion. The use of biodiesel in transportation is critical for reducing greenhouse gas emissions and the European Union’s understanding of the role biofuels must play in the automobile sector, particularly in street transportation, is established. To establish trust, a positive working relationship with all stakeholders, including vehicle manufacturers and oil corporations, was established with the goal of transferring technical know-how, producing fundamental data on cultivation and extraction, marketing strategies, capacity development, coordination, and the execution conduct of necessary research on all the elements of new generation biofuels, and international cooperation.

Because of its long-term viability and environmental benefits, biodiesel derived from vegetable oil is now a viable alternative to petro-diesel (Fernando et al., 2006). Various biodiesel production sources are chosen depending on their accessibility in a given region or country (Sadia et al., 2013). Initially, researchers were interested in edible vegetable oil sources for biodiesel production, such as soya bean, palm, rapeseed, sun flower, canola, and safflower, but these oil sources pose a severe danger to food security (Ghadge and Raheman 2006; Vicente et al., 2004; Kulkarni et al., 2006; Meka et al., 2007). To overcome this obstacle, researchers began investigating the non-edible oil sources in order to develop biodiesel, which proved to be a cost-effective and efficient alternative. Researchers have been already working on a number of nonedible oil sources, such as cotton, rubber, Jatropha, Pongamia, tobacco, and Calophyllum (Kansedo and Lee 2009; Ramadhas et al., 2005; Berchmans and Hirata 2008; Naik et al., 2008; Veljkovic et al., 2006; Sahoo and Das 2009). In this context, the Dodonaea plant was studied quantitatively and qualitatively for biodiesel production, and the results were unexpected.

**FIGURE 1:**
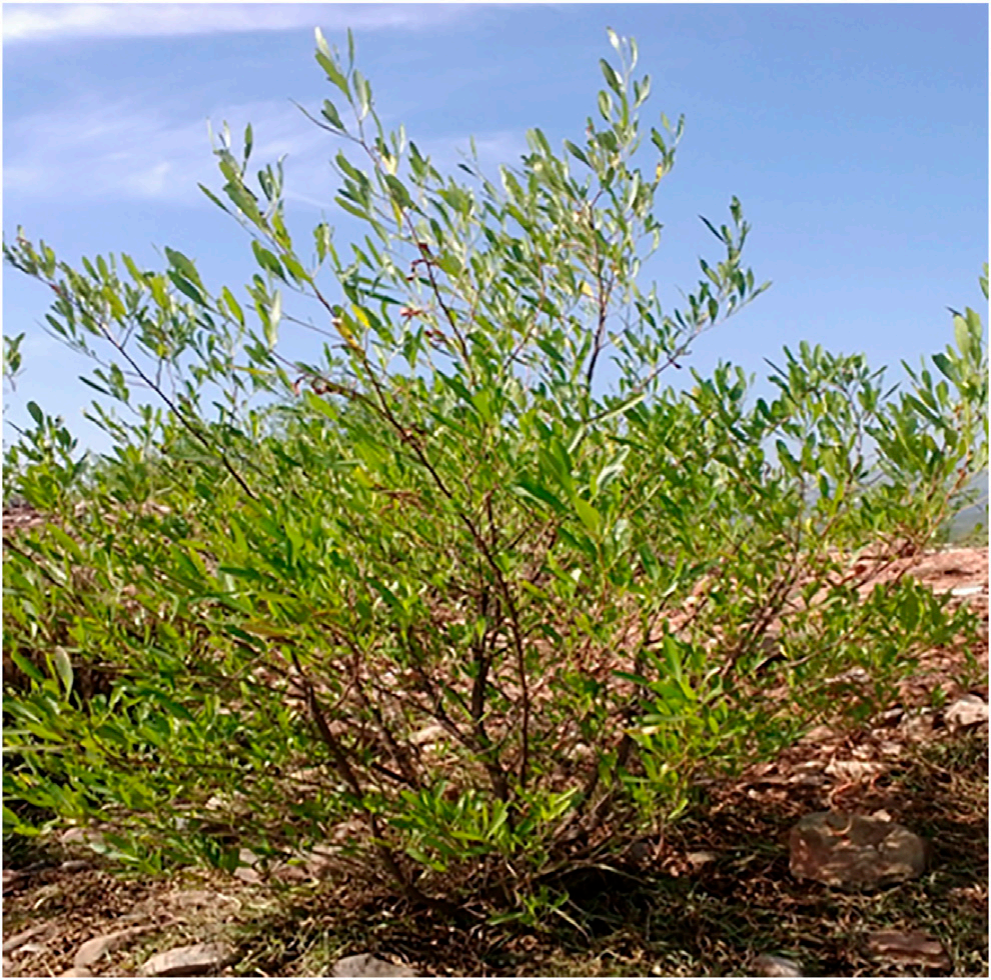
Matured plant of dodonaea (*Dodonaea viscose*).

Dodonaea belongs to the Sapindaceae family (*Dodonaea viscosa* Jacq.). It is an evergreen shrub or small tree that grows throughout the tropics and subtropics of the world from the coast to an altitude of more than 2000 m, as shown in Figure 1. It is an Australian native, but it can also be found in New Zealand, Mexico, Florida, Africa, the Virginia Islands, Arizona, South America, and India. In Pakistan’s subtropical regions, Dodonaea is prolific, generating a dense population (Venkatesh et al., 2008; Rani et al 2009; Barkatulla and Ibrar 2010; Prakash et al., 2012; Lawal and Yunusa 2013).

The therapeutic benefits of Dodonaea are well-known. The locals utilize its leaves, blossoms, and roots to treat a number of ailments, including skin infections, fever, pain, swelling, diarrhea, toothache, and headache (Cribb and Cribb 1981; Rojas et al., 1996). Dodonaea leaves are used to cure bone fractures because they have antibacterial, anti-inflammatory, antifungal, antiulcer, antihyperglycemic, antidiarrheal, antimicrobial, antioxidant, analgesic, and antipyretic qualities (Prakash et al., 2012). In India, it is used as a wood fuel source, and the seeds are used as a fish poison (Jain and Singh 1999; Wagner et al., 1987; Parihar and Dutt 1947). The seeds contain 20.23% fixed oil, according to the literature. However, due to their poisonous nature, they are not used for cooking or other purposes. Apart from the previous discussion, Dodonaea oil has been proposed for biodiesel production in various industries in this research work.

Many seed-bearing plant species are grown for the purpose of production in the world (Mushtaq et al., 2009), but less systematic data on Dodonaea seed oil and its usage in qualitative biodiesel production are available due to its nonedible qualities. In this work, Dodonaea oil was researched systematically for qualitative and quantitative biodiesel synthesis. Taking into consideration the abovementioned statement, Dodonaea oil is recognized as a low-cost source for the bioenergy industry.

## 2 Materials and Methods

### 2.1 Materials

The taxonomic study and biodiesel synthesis were conducted in the Department of Plant Sciences, Quaid-i-Azam University and Islamabad’s Biofuel & Biodiversity and Anatomy Labs, respectively. The raw materials used in this study were Dodonaea seed, oil, methanol, potassium hydroxide, and anhydrous sodium sulfate. All the reagents and chemicals were provided by Merck (Germany), Schrlau (Spain), and Sigma-Aldrich and were used without any purification.

### 2.2 Oil Extraction

**FIGURE 2:**
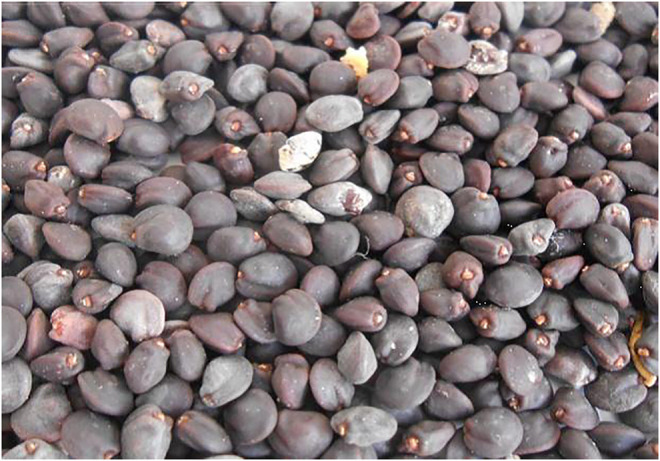
Seed sample of Dodonaea plant species.

Dodonaea seeds were purchased from a local agriculture sale (Figure 2). The oil was extracted from the oven-dried seeds using a mechanical oil expeller (KEK P0015, 10127 Germany). The oil was extracted and processed before being utilized to make biodiesel. To remove contaminants, the crude oil was filtered *via* Whatman filter paper. For further testing, the filtered oil was stored in glass bottles and kept at room temperature (Hussain and Mezan 2010).

### 2.3 Biodiesel Synthesis

#### 2.3.1 Experimental Setup

Biodiesel was made from Dodonaea crude oil using the alkaline transesterification mechanism. For the transesterification procedure, a 250-mL conical flask, a multiple heating magnetic stirrer (Am4, VELP SCIENTIFICA), and a thermometer were used.

#### 2.3.2 Alkaline Transesterification

On a hot plate, the filtered oil was heated to 120°C to remove any moisture and then cooled to 60°C. KOH was dissolved in methanol and stirred for 30–35 min to produce methoxide. At 60°C, methoxide was added to the oil and swirled for 60 min. The mixture was allowed to settle for 8–10 hrs or overnight at room temperature after an hour. Three lyres were discovered in the end. In the base of crude biodiesel, a gelatinous material known as glycerin was produced in the shape of tiny spots floating on the surface. The lyres were isolated using simple handling devices. The crude biodiesel was rinsed in warm water to remove the contaminants. The method was performed two to three times to remove all the contaminants. Anhydrous sodium sulfate was added after washing to remove any leftover moisture. The biodiesel was distilled after purifying it and rotated at 55°C for 1 h to remove any extra methanol.

### 2.4 Optimization of Biodiesel

A number of transesterification processes were carried out with the changing oil-to-methanol molar ratios, different kinds of catalyst concentrations, reaction time, and temperature to determine the best conditions for the maximum conversion of oil to biodiesel.

**FIGURE 3:**
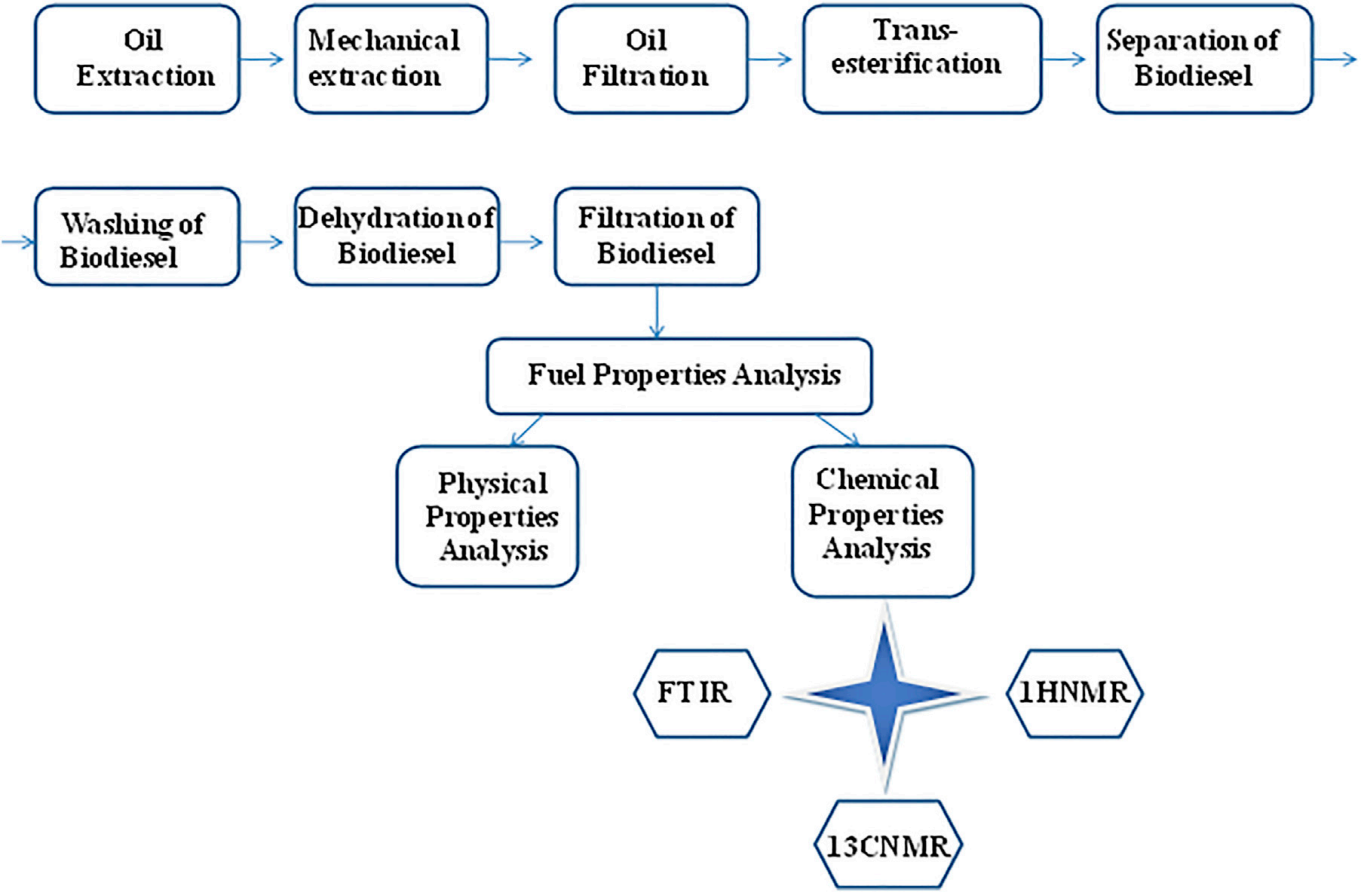
Flow chart of the biodiesel synthesis techniques adopted for experimental work.

The process of the transesterification reaction was carried out in a 1/2 liter three-necked round-bottom flask equipped with a sampling outlet, reflux condenser, thermometer, and magnetic stirrer. Approximately, 250°ml dodonaea filtered oil was heated. The temperature was maintained up to 120°C for 1 h. The moisture and degraded mono, di-glyceride were removed from the acylglycerole. The transesterification reaction of dodonaea oil was carried out with various oil molar ratios and catalyst concentrations (w/w). The temperature (60°C), reaction time (2 h), and stirring velocity (600 rpm) were kept constant for the reactions. The resultant product after the complete reaction was allowed to cool down at room temperature. The upper phase contained a thin spot of soap and the middle part biodiesel, while the base phase contained a gelatinous mass of glycerin, and the mixture was separated by simple decantation. The main dogma of biodiesel preparation is presented in the flow chart of the biodiesel (Figure 3).

In the end, the mixture was separated into two layers: the upper layer contain dodonaea crude biodiesel having an excess amount of methanol. The crude biodiesel was purified by residual methanol distillation at 65°C for 60 min by a moderate rotary evaporator. The remaining catalyst together with other inorganic impurities formed soap, and some catalyst was removed by consecutive washing steps with distilled water by adding three to four drops of weak acid (CH_3_COOH) to neutralize the remaining catalyst. The extra water molecule was removed with the help of anhydrous sodium sulfate (Na_2_SO_4_) followed by filtration (Ullah et al., 2014; Ullah et al., 2015a).

### 2.5 Determination of Biodiesel Fuel Properties Analysis

This article also looks at the qualities of the prepared biodiesel fuel. Quantitatively, the fuel properties test such as color, flash point, density, kinematic viscosity, pour point, cloud point, cetane number, sulfur content, and acid number of the prepared biodiesel sample in contrast to petro-diesel were analyzed and matched with the American Society for Testing and Materials (ASTM) standards (Ullah et al., 2014; Ullah et al., 2015b; Ullah et al., 2015c).

### 2.6 Profiling and Characterization of Biodiesel Using FT-IR and NMR Spectroscopy

**FIGURE 4:**
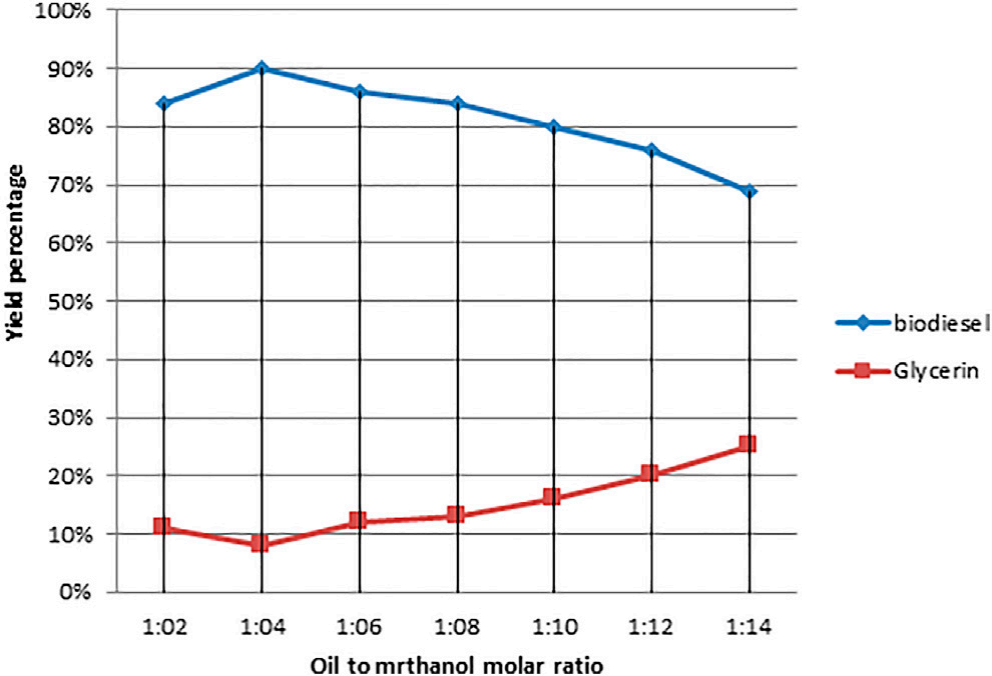
Effect of variation in the molar ratio of oil to alcohol on biodiesel production yield.

The biodiesel that had been prepared was profiled and classified using analytical experiments. FT-IR spectroscopy (Perkin Elmer-TENSOR27) in the 6000–600 cm^−1^ region was used to monitor the reaction. NMR spectroscopy, which includes ^1^H NMR and ^13^C NMR, was used to determine the maximal conversion and presence of proton and carbonyl carbon groups in the synthesized biodiesel (Figure 4 and Table 1).

**TABLE 1:** Fuel property analysis of the Dodonaea biodiesel.

Fuel properties	Method	Results	ASTM
Colour	ASTMD-1500	2	2.0
Flash point °C (PMCC)	ASTM D-93	102	60–100
Density @15°C Kg/L	---------------	0.863	0.86–0.90
K. Viscosity 40°C cSt	ASTM D-445	3.97	1.9–6.0
Pour point °C	ASTM D-97	−12	−15 to 16
Cloud point °C	ASTMD-2500	−15	−3 to 12
Sulfur % wt	ASTMD-4294	0.0043	0.05
Total acid no. mg/KOH/gm	ASTM D-974	0.73	0.5

## 3 Results and Discussion

### 3.1 Oil Contents

The presence of 20.27% oil content in Dodonaea seeds has been documented in the literature (Parihar and Dutt 1947), but the percentage of oil expression present in Dodonaea seeds was 23% using the Soxhlet operations. Oil with a concentration of more than 20% is regarded as suitable for biodiesel processing, according to the research. Due to its low cost and accessibility, Dodonaea oil is within the proposed margin and may be used simply for biodiesel production (Fernando et al., 2006).

### 3.2 Conversion of Dodonaea Seed Oil to Fatty Acid Methyl Esters

Dodonaea oil can be converted to fatty acid methyl esters (FAMEs) using a variety of methods. In our current research, we performed transesterification reactions. Transesterification with potassium hydroxide is used to achieve the best output of biodiesel (Karmaker et al., 2010). The main goal of the research was to see if it was possible to convert Dodonaea oil to biodiesel under perfect circumstances for maximum yield. Because of its short chain and quick conversion time, methanol was utilized to repair the reaction. Before the transesterification process, the oil was heated to 120°C and then cooled to 60°C at room temperature.

Moreover, methoxide is produced by dissolving KOH in methanol and stirring for 30–35 min at 600 rpm (Mushtaq et al., 2009). The resulting biodiesel production is higher when methoxide is used instead of hydroxide catalyst pellets. At 65°C, methoxide was added to the oil and agitated for an hour, allowing the reaction mixture to settle and isolate the newly formed chemical. The mixture was allowed to sit for 8–10 h. The results revealed two noticeable lyres and a few tiny soap spots on the surface. The layer base was built of gelatinous glycerin and crude biodiesel. A maximum yield of the biodiesel ( 90%) was reached using proton NMR spectroscopy.

### 3.3 Effect of Variables on Biodiesel Production

**FIGURE 5:**
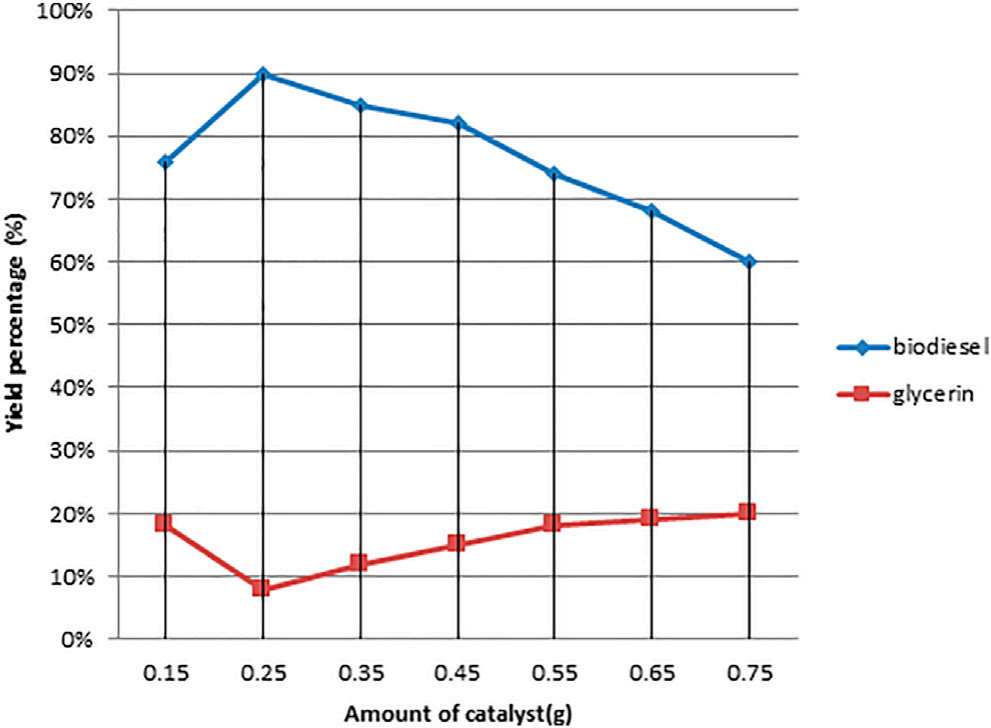
Effect of variation in catalyst (KOH) concentration on the Dodonaea biodiesel production.

**FIGURE 6:**
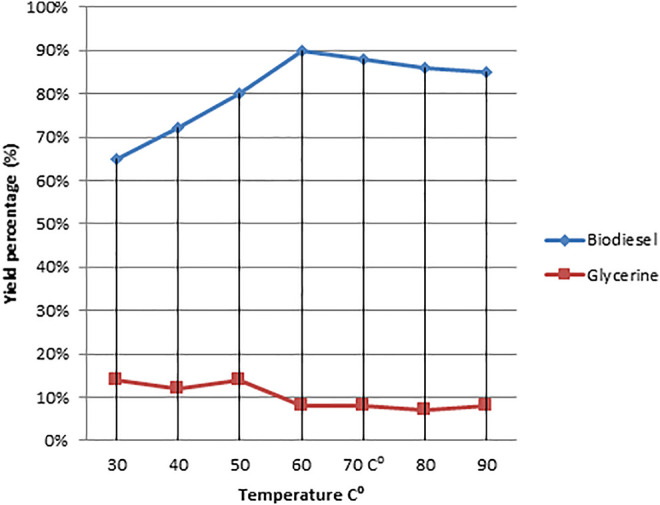
Effect of temperature variation on the Dodonaea biodiesel production yield.

**FIGURE 7:**
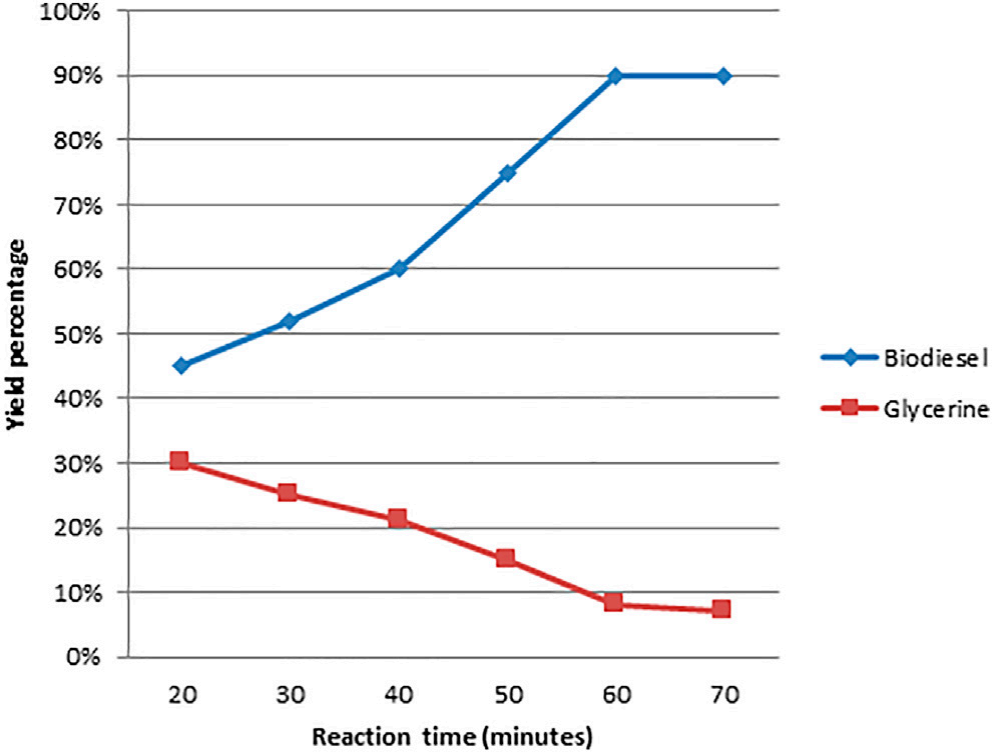
Effect of reaction time on the production of biodiesel from Dodonaea.

**FIGURE 8:**
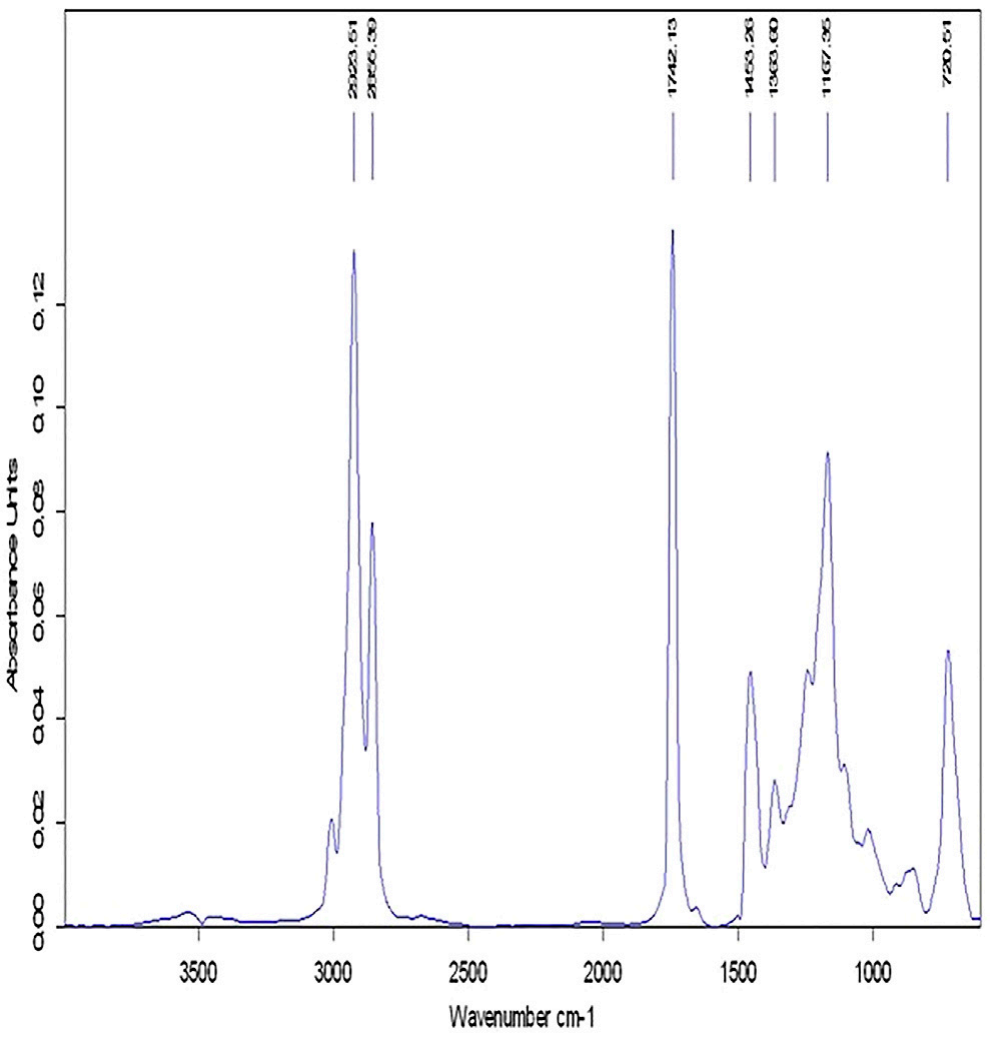
FT-IR spectrum showing various peaks in the Dodonaea prepared biodiesel.

As illustrated in Figures 5–8, a series of tests were conducted in order to obtain the best output of biodiesel from non-edible Dodonaea oil. The impacts of the following parameters were explored and their ideal circumstances.

#### 3.3.1 Effect of Molar Ratio of Oil to Methanol

The molar ratio of oil to methanol in the transesterification process is an important factor that determines the yield of methyl ester (Lin et al., 2009). In the following transesterification operations, the oil-to-methanol ratio was changed as follows: The rest of the parameters were held constant during the reaction: 1:4, 1:6, 1:9, 1:12, 1:15, and 1:18. Table 1 demonstrates that at 1:6 M ratios, the greatest conversion of triglycerides to methyl esters occurred, with a yield of 90%. At 1:6 oil-to-methanol molar ratio, Freedman found a 90–97% conversion of triglycerides to methyl esters, which is similar to our findings (Freedman et al., 1984). According to Freedman’s observations, increasing the molar ratio of oil to methanol initially increases the conversion percentage, but further increasing the molar ratio of oil to methanol reduces the conversion of glycosides to methyl esters (Patil and Deng 2009; Sharma and Singh 2010).

#### 3.3.2 Effect of Catalyst Concentration on Conversion

In the oil with the freest fatty acids, the use of a base catalyst outperforms the use of an acid catalyst, and potassium hydroxide outperforms sodium hydroxide in the transesterification reaction. Moreover, because of the emulsion and soap formation, if the free fatty acid level is higher than 3%, the final product will not contain biodiesel (Fukuda et al., 2001).

To generate the largest yield of biodiesel, the basic catalyst potassium hydroxide was chosen for transesterification in this investigation (Shahid and Jamal 2011). In the extraction of glycerol from crude biodiesel, potassium hydroxide outperformed sodium hydroxide, according to Encinar et al. (Encinar et al., 2005). Other researchers converted Turkish safflower oil to biodiesel using potassium hydroxide, attaining a biodiesel output of 97.7%. (Isigigur et al., 1994).

To convert Dodonaea oil to biodiesel, a base catalyst was used in the transesterification reactions. Different potassium hydroxide concentrations were utilized in different processes (0.15, 0.25, 0.35, 0.45, 0.55, 0.65, and 0.75). The maximum conversion percentage of the biodiesel was achieved at 90 percent using 0.25% potassium hydroxide. The potassium hydroxide concentration continuously rises as a result of saponification, lowering the conversion percentage. Because of emulsion formation, utilizing a greater concentration of potassium hydroxide catalyst in the transesterification reaction has a detrimental impact on methyl ester yield and consistency (Figure 6) (Meher et al., 2006; Rashid et al., 2008).

#### 3.3.3 Effect of Temperature on Conversion

Transesterification can occur at a variety of temperatures depending on the oil used in the reaction (Ma and Hanna 1999). The lowest temperature range has a significant impact on the rate of transesterification reaction and, ultimately, the end product. The temperature increases the lower oil viscosity proportionally and directly increases the reaction rate because the reaction receives more energy (Koh and Ghazi 2011). Saponification was accelerated until alcoholysis was completed if the reaction temperature was above the boiling point of methanol (Dorado et al., 2004). The influence of temperature on fatty acid methyl ester yield from Dodonaea oil during the transesterification reaction was investigated using different temperatures (30, 40, 50, 60, 70, 80, and 90°C). The ideal temperature range for these reactions was discovered to be 60–65°C.

According to the data, increasing the temperature to 60°C boosted the biodiesel yield significantly, as shown in Figure 7. The production of biodiesel decreased as the temperature rose. The best temperature for the optimal conversion of cotton seed oil to biodiesel, according to Rashid et al., is 65°C (Rashid et al., 2008). At 60°C, Pongamia, Jatropha, and sesame all had the highest biodiesel yields, with Jatropha having the maximum yield of 99% (Mushtaq 2009). It was observed that the highest yield of biodiesel could be attained at room temperature by simply increasing the reaction time. As illustrated in Figure 6, the maximum temperature has an impact on the saponification reaction, resulting in a low biodiesel output (Reference Deleted by mistake).

#### 3.3.4 Effect of Reaction Time on Conversion

The length of the transesterification reaction has a significant impact on the production of biodiesel. They must be swirled well at a consistent rate to achieve complete contact between the catalyst and the triglycerides during the transesterification reaction. A series of tests were carried out using Dodonaea seed oil to assess the effect of reaction time on biodiesel yield. The reaction time was increased from 20 to 70 min by including a 10-minute break. A response time of 60–70 min was determined to be optimal, resulting in a biodiesel yield of 90%. As the reaction temperature was raised, the production of biodiesel increased, as illustrated in Figure 8. (Mushtaq and colleagues, 2009).

### 3.4 Biodiesel Characterization

Color, flash point, viscosity, density, pour point, cloud point, sulfur percentage, and acid quantity were all examined in the biodiesel generated from Dodonaea plant oil. The fuel characteristics of Dodonaea biodiesel were investigated and compared to those of ASTM in Table 1.

#### 3.4.1 Flash Point

When handling, storing, and transporting fuel, the flash point is an important factor to consider. This is the temperature at which the biodiesel can ignite when exposed to flame. A greater flash point is generally regarded to reduce the risk of fire (Syam et al., 2009; Krisnangkura and Simamaharnop). The biodiesel’s key advantage over petroleum-based fuel is its flash point (Anwar et al., 2010). In this study, the flash point of Dodonaea biodiesel was estimated using ASTM D-93 and was found to be 102°C. This fuel has a greater flash point than the regular petro-diesel, indicating that it is safe for use in transportation.

#### 3.4.2 Density

According to Table 1, the density of Dodonaea biodiesel @150°C kg/L was found to be 0.863 kg/L in this investigation using the ASTM D-1298 procedure. The density of Dodonaea biodiesel was found to be higher than that of petroleum diesel, but still within the ASTM criteria.

#### 3.4.3 Kinematic Viscosity

The viscosity of a fuel influences its atomization upon injection into the combustion chamber and the development of soap and engine deposits (Knothe and Steidley 2005). Viscosity, which appears to oppose any dynamic change in fluid motion, is used to quantify the internal fluid friction of the fuel to flow. Injector lubrication and atomization are affected by the viscosity of the fuel.

Low-viscosity fuels do not offer adequate lubrication for fuel injection pumps to fit perfectly, causing leakage or increased wear. If the viscosity is low, the leakage will result in engine power loss. If the viscosity is high, the injection pump will be unable to supply enough gasoline to fill the pumping chamber, resulting in engine power loss once more. Kinematic viscosity (1.9–6.0 mm2/s in ASTM D 6751 and 3.5–5.0 mm^2^/s in EN 14214) is one of the biodiesel specifications [49]. As indicated in Table 1, the kinematic viscosity of Dodonaea biodiesel was tested using ASTM D-445 at 40°C and was determined to be 3.97 mm^2^/s. The measured value fell within the ASTM D-445 standard range (1.9–6.0 mm2/s). The lower viscosity of the biodiesel makes it easier to pump and atomize (Knothe and Steidley 2005).

#### 3.4.4 Pour Point

The pour stage is the lowest temperature at which a fuel can pour or flow when cooled under particular conditions. A high pour point is frequently associated with poor fuel characteristics [47]. The pour point of methyl ester generated from Dodonaea oil according to ASTM D-97 was determined to be −12°C, which is nearly comparable to the pour point of Pongamia [44]. This value is within the ASTM biodiesel requirement ranges. The type of fatty acid branched chain contained in the original oil has an impact on the pour point (Lee et al., 1995).

#### 3.4.5 Cloud Point

When gasoline is refrigerated under certain conditions, the cloud point is the temperature at which paraffin (wax) crystallizes. A fuel’s high cloud point also indicates that it has poor characteristics. In this study, the cloud point of Dodonaea methyl ester, as determined by ASTM D-2500, was found to be −15°C (Table 1).

#### 3.4.6 Sulfur Content

The sulfur concentration was evaluated using ASTM D-4294 in this study. The biodiesel generated from Dodonaea oil has an extremely low sulfur level of 0.0043%, as reported in Table 1. The range of sulfur is less than 1 ppm, unlike the normal petro-diesel fuel, which has a sulfur level of 50 ppm. The fact that biodiesel has a sulfur percentage of less than 1 ppm is a major benefit to both engine life and the environment. The low sulfur content of the biodiesel makes it a good fuel for extremely polluted areas as SO_2_ levels in biodiesel are substantially lower than in conventional diesel. Traditional diesel has a lower pour point, flash point, and sulfur content. Biodiesel has a greater pour point, flash point, and sulfur content (Anwar et al., 2010).

#### 3.4.7 Acid Number

The total acid number is the amount of potassium hydroxide required to neutralize the free fatty acids in biodiesel (Arjun et al., 2008). The most straightforward method for determining fuel content is to examine the acid value. The total acid number of Dodonaea biodiesel was estimated using ASTM D-974 and was found to be 0.73 mg KOH/gm (Table 1). As the acid value increases, the rubber components of the older engine fuel supply systems degrade.

### 3.5 Biodiesel Chemistry

The current research study used analytical methods to analyze Dodonaea biodiesel. The conversion of Dodonaea oil to methyl esters was validated using these methods. The functional group and conversion percentage were determined using ^1^H and ^13^C NMR, respectively, and the transesterification reaction was tracked using FT-IR.

#### 3.5.1 FT-IR Study

Fourier-transform infrared spectroscopy was used to identify the functional groups and peaks corresponding to distinct stretching and bending vibrations in the Dodonaea biodiesel. The production of fatty acid methyl esters was confirmed by the FT-IR measurements.

**FIGURE 9:**
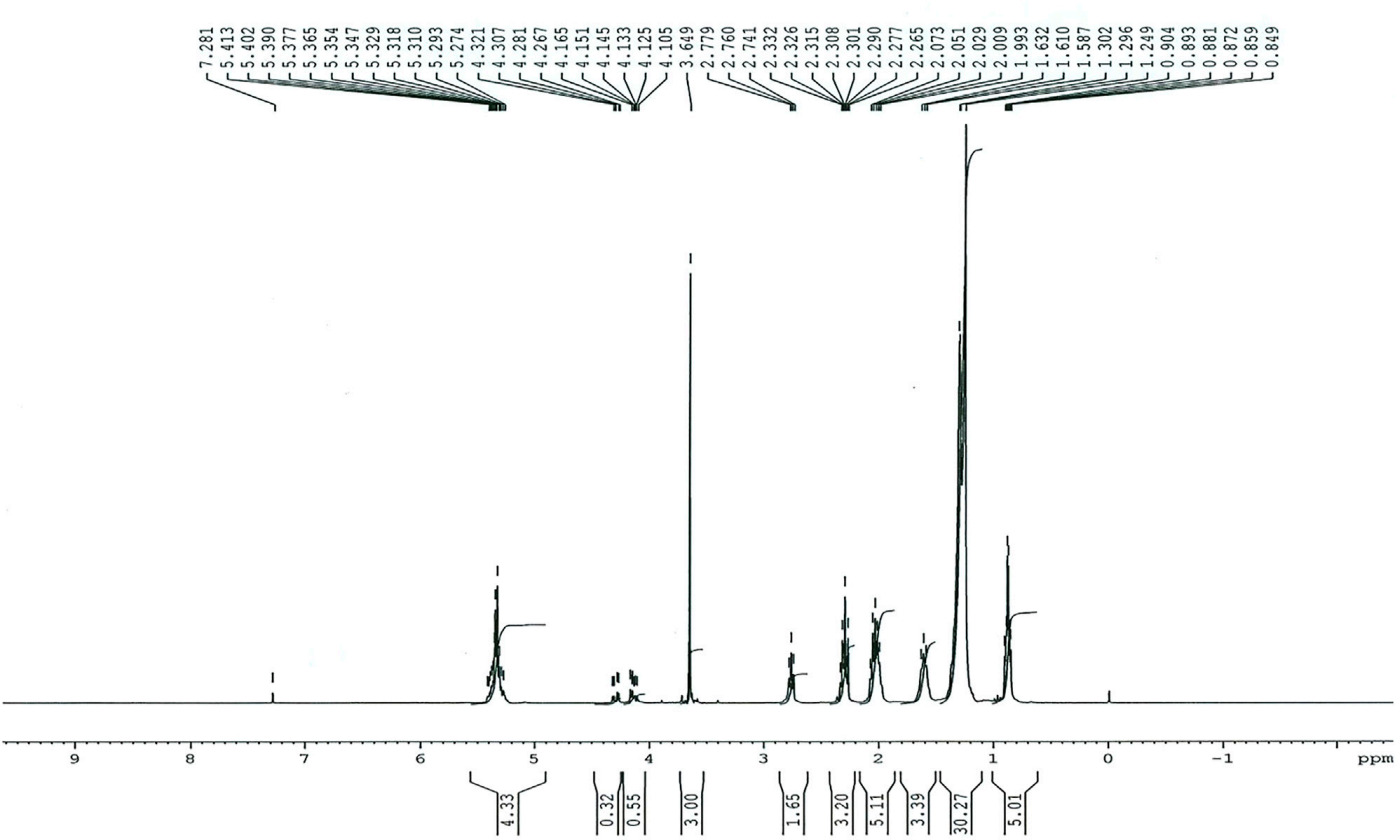
^1^H NMR spectrum showing the chemical bonding in the Dodonaea plant prepared biodiesel.

FT-IR analysis, which is created by transesterification from their peaks, also confirms different functional groups (Knothe 1999). This study approach can also be utilized to assess the oil content in the adulterated biodiesel–petrodiesel mixture with minor modifications (Mahamuni and Adewuwi 2009). There were two primary distinctive bands for detecting methyl esters: one was carbonyl carbon, which had a peak at 1735–1750 cm^−1^, and the other was C-H, which had a peak at 2850–3000 cm^−1^. (Figure 9). The carbonyl carbon (C=O) and alkane (C-H) peaks of the ester occurred at 1742.13 cm^-1^ and 2923.51 cm^−1^, respectively. As demonstrated in Table 2, the alkyl halide peak was at 1167.36 cm^−1^, while the C-H rock peak was at 720.1 cm^−1^. In the same way, the C-H bending peak was found at 1453.26 cm^−1^. The place of the carbonyl group in FT-IR is sensitive to substituent effects and to the structure of the molecule (Bianchi et al., 1995).

**TABLE 2:** FT-IR spectroscopy showing various peaks in the Dodonaea biodiesel.

Peaks	Frequency range (cm^−1^)	Bond	Functional group
2923.51	3000–2850	C-H stretch	Alkanes
1742.13	1750–1735	C=O stretch	Ester, saturated aliphatic
1453.26	1470–1450	C-H bend	Alkanes
1167.36	1300–1150	C-H Wag (−CH_2_X)	Alkyl halide
720.51	725–720	C-H rock	Alkanes

#### 3.5.2 NMR Study

##### *3.5.2.1* ^*1*^ *H NMR Spectroscopy*

**FIGURE 10:**
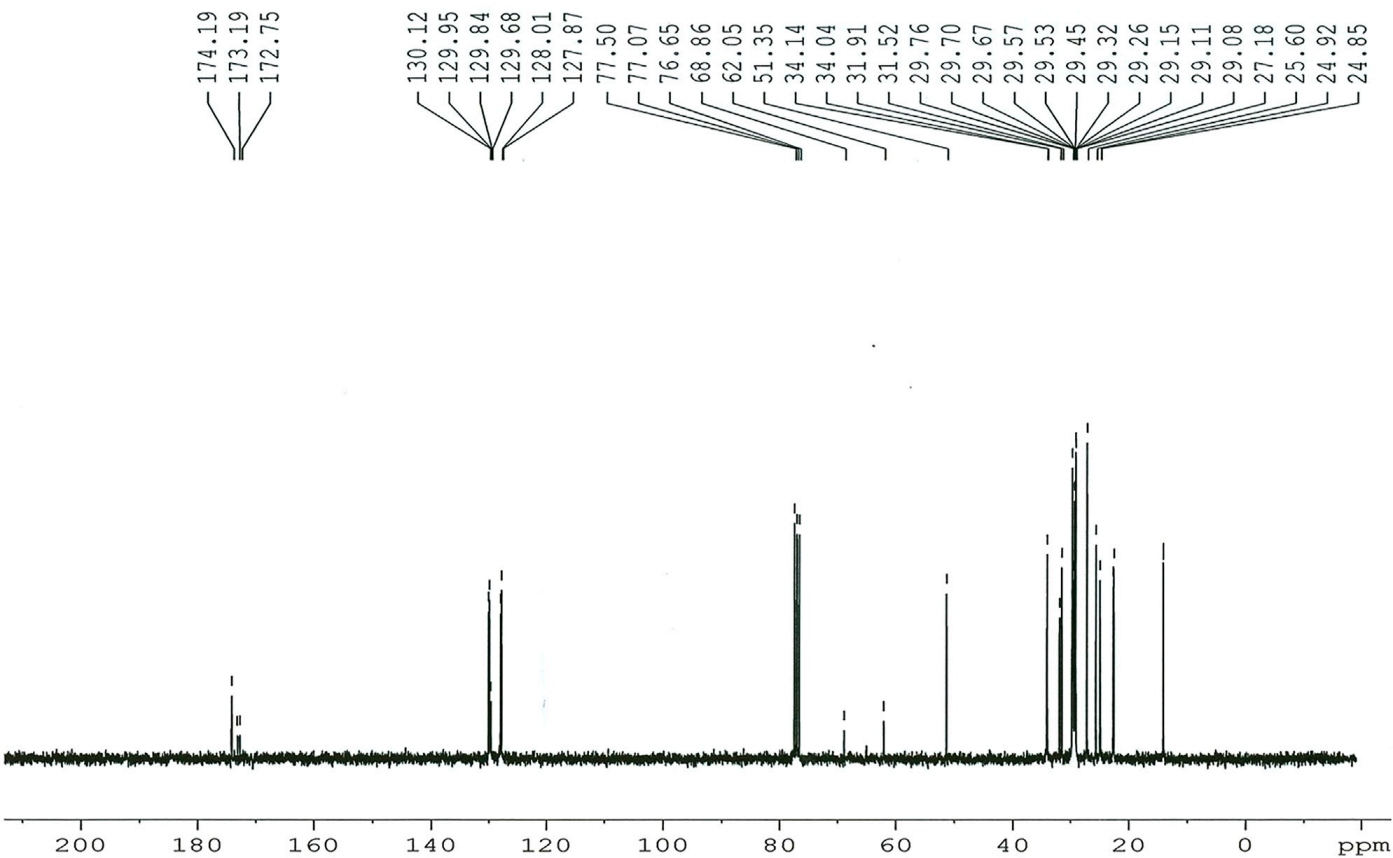
^13^C NMR spectrum of the Dodonaea biodiesel.

^1^H NMR was used to assess the yield of Dodonaea biodiesel (Figure 9). Proton nuclear magnetic resonance spectroscopy was recently used to study the kinetics and product distributions of the transesterification reactions (alcoholysis) between vegetable oils and alcohols (^1^H NMR). Because a tiny aliquot of the batch reaction may be collected at any time and the ^1^H NMR spectrum analysis offers extensive details about the chemical species participating in the reaction, using ^1^H NMR to monitor a reaction is simple and quick (Morgenstern et al., 2006). Using ^1^H NMR spectroscopy, the protons in the alcohol moiety of the resulting methyl esters and the protons of the methylene group next to the ester moiety in TAG were used to track the yield (Ullah et al., 2014). The ^1^H NMR spectra of the methyl ester product obtained by the transesterification of Dodonaea oil are shown in Figure 10. The methoxy proton’s distinctive peak was found at 3.649 ppm. The methylene triplet was discovered at 2.332 ppm. These two peaks at 3.649 and 2.332 ppm demonstrate the presence of methyl esters in the Dodonaea biodiesel. At 1.249 ppm, a strong singlet was detected, and at 0.904 ppm, a peak for the terminal methyl proton was detected. The signal at 5.274 indicates the existence of carbonyl methylene protons and olefinic hydrogen. ^1^H NMR was also used to validate the conversion percentage of triglycerides to the biodiesel using equation 1. (Gelbard et al., 1995; Knothe 2009).

C = 100 × 2AMe/3ACH_2 -------------------------------_ *Eq. (1)*
C = Percentage conversion of triglycerides to the corresponding methyl esters.
AMe = Integration value of the methoxy protons of the methyl esters.
ACH_2_ = Integration value of the α-methylene protons.

##### *3.5.2.2* ^*13*^ *C NMR Spectroscopy*

The presence of carbonyl esters (-COO-) and the C-O group in the Dodonaea biodiesel was determined using ^13^C NMR. The distinctive peaks at 130.12 and 127.87 ppm in ^13^C NMR spectra (Figure 10) indicate the existence of unsaturation. Methylene esters in the Dodonaea biodiesel have unsaturation bonds of 131.92 and 127.10 ppm [56–58]. The peaks at 24.85 and 34.14 ppm correspond to the terminal carbon of the methyl group and the methylene carbon of the long chain, respectively.

## Conclusion

According to the current study on Dodonaea plant oil, this plant provides a promising and innovative source for biodiesel manufacturing. When the following criteria were met, the biodiesel output was reported to be 90% in this study: A 1:6 M oil-to-alcohol ratio, a temperature of 600°C, a reaction period of 70 min, and a concentration of 0.25% potassium hydroxide catalyst was used. The fuel characteristics of the Dodonaea plant biodiesel were analyzed and compared to the American Society for Testing and Materials standards. Dodonaea plant oil is nonedible and a promising source for biodiesel production, based on the fuel qualities and physico-chemical analyses outlined previously. It is also suggested that Dodonaea plant species be grown readily on the marginal and barn land to increase the supply of feedstock for the bioenergy industry. The main theme of various studies and the fuel properties test in the Dodonaea plant oil biodiesel was to monitor and control the quantity and quality of the prepared biodiesel and its potential for reliable commercialization.

## Data Availability

The original contributions presented in the study are included in the article/Supplementary Material, Further inquiries can be directed to the corresponding author.
